# Exercise Training Ameliorates Renal Oxidative Stress in Rats with Chronic Renal Failure

**DOI:** 10.3390/metabo12090836

**Published:** 2022-09-04

**Authors:** Seiko Yamakoshi, Takahiro Nakamura, Lusi Xu, Masahiro Kohzuki, Osamu Ito

**Affiliations:** 1 Division of Clinical Pharmacology and Therapeutics, Tohoku University Graduate School of Pharmaceutical Sciences, Sendai 980-8576, Japan; 2 Department of Internal Medicine and Rehabilitation Science, Tohoku University Graduate School of Medicine, Sendai 980-8574, Japan; 3 Division of Integrative Renal Replacement Therapy, Tohoku Medical and Pharmaceutical University, Sendai 983-8536, Japan; 4 Division of General Medicine and Rehabilitation, Faculty of Medicine, Tohoku Medical and Pharmaceutical University, Sendai 983-8536, Japan

**Keywords:** exercise, chronic renal failure, oxidative stress, NADPH oxidase, xanthine oxidase

## Abstract

In patients with chronic kidney disease, exercise training with moderate intensity protects renal function and improves mortality. However, the mechanisms of the renal protective effects of exercise training in chronic kidney disease have not been clarified. This study investigated the effects of exercise training on renal NADPH oxidative and xanthine oxidase, which are major sources of reactive oxygen species, in rats with chronic renal failure. Six-week-old, male Sprague–Dawley rats were divided into the sham operation, 5/6 nephrectomy (Nx)+ sedentary, and Nx+ exercise training groups. The Nx+ exercise training group underwent treadmill running. After 12 weeks, systolic blood pressure, renal function, malondialdehyde, renal NADPH oxidase, and xanthine oxidase were examined. Nx induced hypertension, proteinuria, and renal dysfunction, and exercise training attenuated these disorders. Although the plasma levels of malondialdehyde were not different among the group, urinary levels were increased by Nx and decreased by exercise training. Renal activity and expression of NADPH oxidase and xanthine oxidase were increased by Nx and decreased by exercise training. These results indicate that exercise training attenuates hypertension and renal dysfunction and ameliorates NADPH oxidase and xanthine oxidase in rats with chronic renal failure, suggesting that the reduction of reactive oxygen species generation may be involved in the renal protective effects of exercise training.

## 1. Introduction

Chronic kidney disease (CKD) has been recognized as a global public health problem with a high rate of morbidity and mortality. Reduced physical activity is associated with high mortality rates in CKD patients [1]. Exercise training with moderate intensity improves renal function and reduces mortality in CKD patients [2,3,4]. We reported that exercise training ameliorated the pathogenesis of CKD, such as hypertension, proteinuria, renal dysfunction, glomerulosclerosis, and interstitial fibrosis in several CKD model rats [5]. However, the detailed mechanisms of the renal protective effects of exercise training have not been fully elucidated.

CKD has an enhanced oxidative stress status, beginning at its early CKD stage due to increased generation of reactive oxygen species (ROS) and decreased antioxidant activity [6,7]. Oxidative stress plays an important role in the development and progression of CKD [8]. Major sources of ROS generation include nicotinamide adenine dinucleotide phosphate (NADPH) oxidase and xanthine oxidase (XO) [9]. The elevated renin-angiotensin system in CKD facilitates NADPH oxidase activity and ROS generation [10]. Rats with chronic renal failure (CRF) induced by 5/6 nephrectomy (Nx) show an increased expression of renal NADPH oxidase subunits [11]. However, effects of exercise training on renal NADPH oxidase or XO have not been reported in Nx-induced CRF rats.

The effects of ROS scavenging and inhibition of ROS generation on blood pressure and renal dysfunction have been reported. The superoxide dismutase mimetic, Tempol, decreased renal oxidative stress but did not affect proteinuria, renal function, or glomerular and tubulointerstitial damage in Nx-induced CRF rats [12]. The NADPH oxidase inhibitor, apocynin, decreased blood pressure but did not affect serum creatinine in Nx-induced CRF rats [13]. Several studies have reported the effects of XO inhibitors on blood pressure and renal function. The XO inhibitor, allopurinol, inhibited the increases in serum creatinine but did not affect blood pressure or proteinuria in CKD patients [14]. Topiroxostat decreased albuminuria without changes in the estimated glomerular filtration rate or blood pressure in CKD patients [15]. Febuxostat attenuated proteinuria, renal dysfunction, and renal interstitial fibrosis but did not affect blood pressure in Nx-induced CRF rats [16]. Febuxostat attenuated hypertension, renal dysfunction, and renal histology in Dahl salt-sensitive rats fed a high-salt diet [17]. These results suggest that the ROS generation by NADPH oxidase and XO may induce blood pressure elevation and renal damages, respectively, and that superoxide dismutase may not affect blood pressure or renal damage in Nx-induced CRF rats.

Exercise training consumes large amounts of oxygen in organs and skeletal muscle, increasing oxidative stress in the process, but appropriate exercise training attenuates ROS generation and increases oxidative stress tolerance [18]. Exercise training decreased serum malondialdehyde (MDA) as an oxidative stress marker in dialysis patients [19]. Thus, we hypothesized that exercise training could suppress renal ROS generation, NADPH oxidase, and XO in CKD patients and models. To elucidate this hypothesis, this study examined the effects of exercise training on renal ROS generation enzymes, NADPH oxidase, and XO, in Nx-induced CRF rats.

## 2. Materials and Methods

### 2.1. Experimental Animals

This study was approved by the Animal Welfare Committee at Tohoku University (2015IDOU-133) and observed the guidelines for animal experiments and related activities of Tohoku University and the guiding principles of the National Institutes of Health.Six-week-old, male Sprague–Dawley rats (Charles River, Yokohama, Japan) were randomly assigned: sham operation (Sham, *n* = 6), 5/6 nephrectomy (Nx)+ sedentary (NxS, *n* = 6), and Nx+ exercise training (NxEx, *n* = 6). Nx was performed as described previously [20]. The rats were housed in an animal care facility at Tohoku University School of Medicine in a controlled room, with a 12 h light–dark cycle. Throughout the experiments, the rats were fed a normal diet (Nosan Corp., Yokohama, Japan) and had free access to tap water. The NxEx group underwent treadmill running (KN-73; Natsume Industries, Tokyo, Japan) at an aerobic intensity (no grade incline, 20 m/min, 60 min/day, 5 days/week) for 12 weeks as previously described [21,22].

### 2.2. Measurements of Blood Pressure and Plasma and Urine Parameter

Body weight and systolic blood pressure were measured every 2 weeks using the tail-cuff method (MK-2000; Muromachi Kikai, Tokyo, Japan). In the final experimental week, all rats were placed in individual metabolism cages, and their urine samples were collected and placed on ice for 24 h.

At the end of the experiments, all rats were anesthetized with 0.15 mg/kg of medetomidine, 2.0 mg/kg of midazolam, and 2.5 mg/kg of butorphanol interperitoneally [23], and blood samples were collected by cannulating the abdominal aorta. Plasma urea nitrogen, creatinine, and urinary protein were measured by the Nagahama Life Science Laboratory (Nagahama, Shiga, Japan).

### 2.3. Measurement of Thiobarbituric Acid–Reactive Substances

Plasma and urinary thiobarbituric acid reactive substances (TBARS) were measured using a colorimetric assay kit (Cayman Chemical, Ann Arbor, MI, USA) [24], and TBARS levels were expressed as malondialdehyde (MDA) concentrations and excretions.

### 2.4. Tissue Sample Preparation

After sacrifice, a portion of the remnant kidney was quickly dissected. The cortical tissues were homogenized in a 0.1mol/L potassium phosphate buffer (pH 7.25) containing 30% glycerol, 1 mmol/L dithiothreitol, and 0.1 mmol/L phenylmethylsulphonyl fluoride. The homogenate samples were centrifuged at 3000× *g* for 15 min, and the supernatant was collected and stored at −80 °C until further analysis.

### 2.5. Measurement of NADPH Oxidase and XO Activities

The NADPH oxidase and XO activities were measured by a lucigenin-enhanced chemiluminescence assay and a pterin based-assay, as described previously [25,26].

### 2.6. Immunoblot Analysis

Protein expression was examined with western blotting, as described previously [26]. Protein concentration of the samples were measured by the Bradford method [27]. The antibodies raised against NADPH oxidase isoform 2 (Nox2) (Santa Cruz Biotechnology, Dallas, TX, USA), NADPH oxidase isoform 4 (Nox4) (Abcam, Trumpington, Cambridge, UK), and XO (Santa Cruz Biotechnology, Dallas, TX, USA). Immunoblots were developed using an enhanced chemiluminescence kit (Super Signal; Thermo Fisher Scientific, Waltham, MA, USA). The relative intensities of the bands were quantified using Image J. The intensities of bands were normalized against β-actin (Sigma-Aldrich, St. Louis, MO, USA) as an internal standard, which was assigned a value of 1.

### 2.7. Statistical Analysis

All data are presented as mean ± SEM. Comparisons of the three groups were analyzed using a one-way ANOVA, followed by a Tukey test. *p* values less than 0.05 were considered statistically significant.

## 3. Results

### 3.1. Body Weight, Systolic Blood Pressure, and Plasma and Urinary Parameters

Table 1 shows the body weight, systolic blood pressure, and plasma and urinary parameters at the end of the experiment. Compared with the Sham group, body weight was significantly reduced by Nx but not affected by exercise training. Nx significantly increased systolic blood pressure, and exercise training significantly inhibited the Nx-increased systolic blood pressure. Nx significantly increased plasma creatinine, urea nitrogen, and urinary protein, and exercise training significantly inhibited these Nx-induced renal dysfunctions.

### 3.2. Measurement of Thiobarbituric Acid–Reactive Substances in Plasma and Urinary

**Figure 1:**
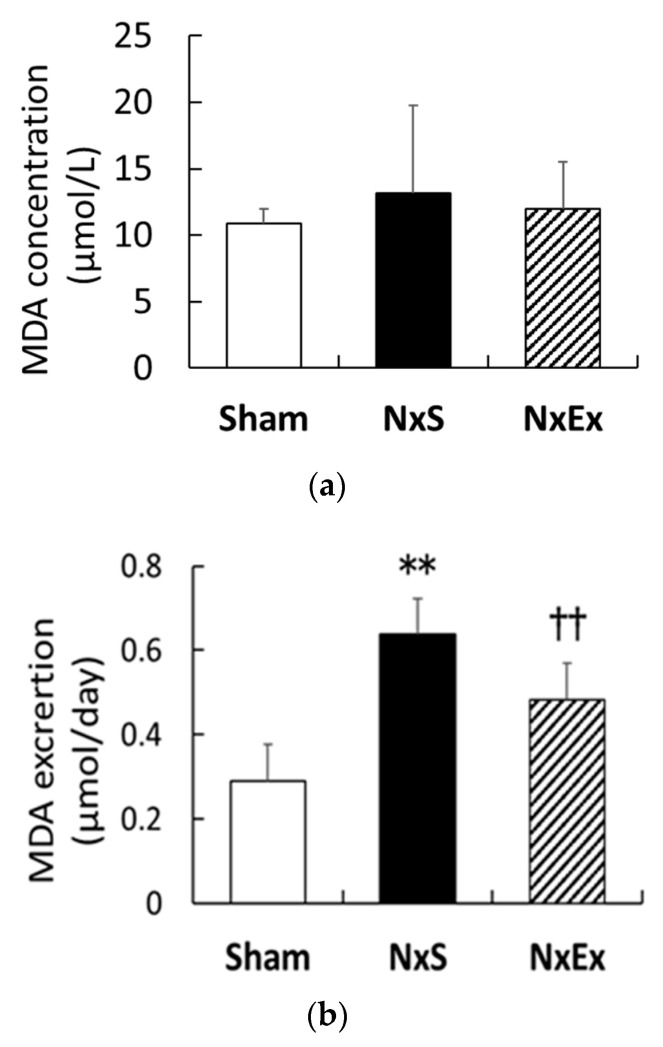
Effects of exercise training on plasma and urinary malondialdehyde (MDA). The plasma MDA concentration (**a**) and urinary MDA excretion (**b**) were compared among the sham operation (Sham), nephrectomy sedentary (NxS,), and nephrectomy exercise (NxEx) groups. Values are the means ± SEM. ** *p* < 0.01 compared with the sham group, ^††^ *p* < 0.01 compared with the NxS group.

Plasma and urinary MDA levels are shown in Figure 1a,b. There were no significant differences in plasma MDA concentrations among the groups (Figure 1a). Compared with the Sham group, Nx significantly increased urinary MDA excretion (0.29 ± 0.038 vs. 0.64 ± 0.03 µmol/day, *p* < 0.01), whereas exercise training significantly inhibited the Nx-increased urinary MDA excretion (0.48 ± 0.039 µmol/day, *p* < 0.01).

### 3.3. NADPH Oxidase and XO Activities

**Figure 2:**
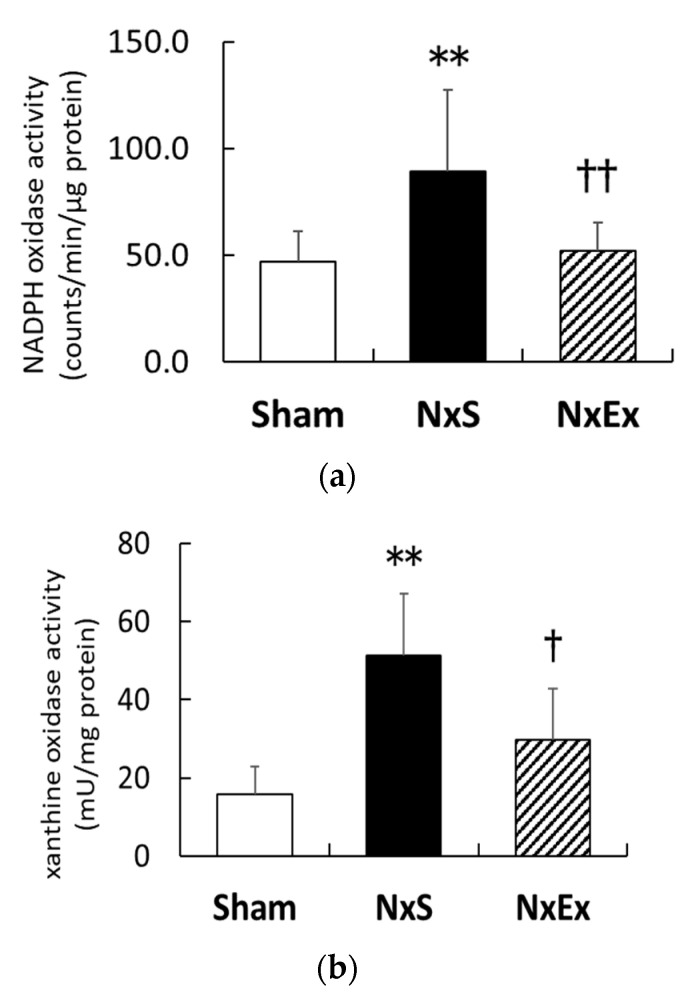
Effects of exercise training on the source of reactive oxygen species. The renal activities of nicotinamide adenine dinucleotide phosphate (NADPH) oxidase (**a**) and xanthine oxidase (XO) (**b**) were compared among the sham operation (Sham), nephrectomy sedentary (NxS), and nephrectomy exercise (NxEx) groups. Values are the means ± SEM. ** *p* < 0.01 compared with the Sham group, ^†^ *p* < 0.05, ^††^ *p* < 0.01 compared with the NxS group.

Compared with the Sham group, Nx significantly increased the renal NADPH oxidase activity (46.8 ± 5.5 vs. 89.0 ± 11.5 count per minute/µg protein, *p* < 0.01), whereas exercise training significantly inhibited the Nx-increased NADPH oxidase activity (52.1 ± 4.8 count per minute/μg protein, *p* < 0.01) (Figure 2a). Nx also significantly increased the XO activity (15.4 ± 2.6 vs. 42.9 ± 4.4 mU/mg protein, *p* < 0.01), and exercise training significantly inhibited the Nx-increased XO activity (27.9 ± 3.7 mU/mg protein, *p* < 0.05).

### 3.4. Protein Expression of NADPH Oxidase Isoforms and XO

**Figure 3:**
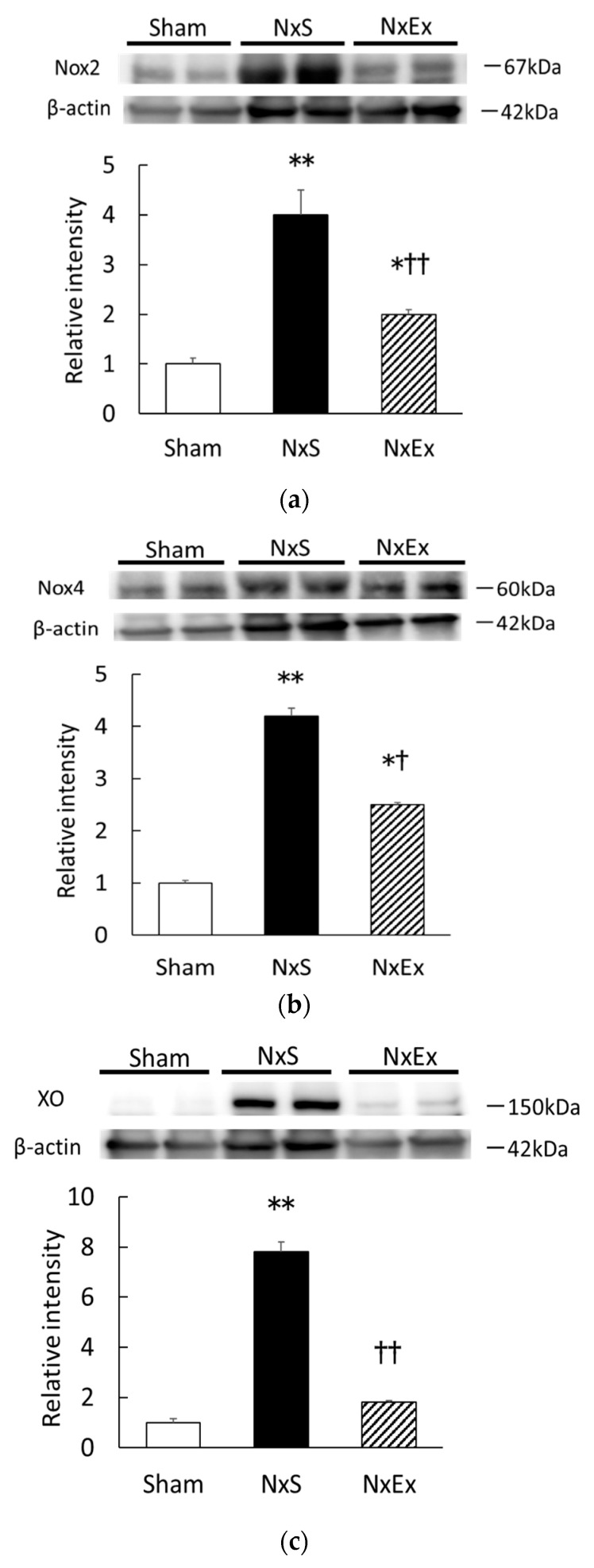
Effect of exercise training on the renal expression of NADPH oxidase 2 (Nox2), NADPH oxidase 4 (Nox4), and xanthine oxidase (XO). Renal cortical expressions of the Nox2 (**a**), Nox4 (**b**), and XO (**c**) were compared among the sham operation (Sham), nephrectomy sedentary (NxS), and nephrectomy exercise (NxEx) groups. The intensities of each protein were normalized against those of β-actin. The intensity of the band in the Sham group was assigned a value of 1. Values are the means ± SEM. * *p* < 0.05, ** *p* < 0.01 compared with the sham group, ^†^ *p* < 0.05, ^††^ *p* < 0.01 compared with the NxS group.

Compared with the Sham group, Nx significantly increased the Nox2, Nox4, and XO expressions by 4.2-, 4.0-, and 7.8-fold, respectively, and exercise training significantly the Nx-increased Nox2, Nox4, and XO expressions 2.5-, 2.0-, and 1.8-fold, respectively. (Figure 3a–c).

## 4. Discussion

This study examined the effects of exercise training on renal oxidative stress, NADPH oxidase, and XO in Nx-induced CRF rats. Exercise training attenuated hypertension and renal dysfunction and inhibited the Nx-increased urinary MDA, renal NADPH oxidase, XO activity, and renal expression of Nox2, Nox4, and XO. This study first revealed the effectiveness of exercise training on renal NADPH oxidase and XO in Nx-induced CRF rats.

Previous studies have indicated renal oxidative stress and its association with renal dysfunction in Nx-induced CRF rats [11,28]. Consistent with these findings, Nx increased urinary MDA excretion, and exercise training attenuated Nx-increased MDA excretion. We further examined the effect of exercise training on the activity and expression of NADPH oxidase and XO in the kidneys. Exercise training decreased the Nx-increased NADPH oxidase activity and the expression of Nox2 and Nox4. The present results are consistent with those of Kim et al., who reported that the renal expression of Nox2 and Nox4 increased in Nx-induced CRF rats [11]. NADPH oxidase is composed of two isoforms: Nox2 and Nox4. Nox4 is highly expressed in the kidney and generates ROS [29]. Renal Nox4 expression is elevated in kidney diseases, including diabetic nephropathy and hypertensive nephropathy, and it contributes to the redox process in various CRF models [30]. Further, upregulation of renal NADPH oxidase via the renin–angiotensin system results in the ROS generation, which contributes to renal fibrosis [31,32]. We previously reported that exercise training inhibited the Nx-activated renal renin–angiotensin system and renal fibrosis [22]. The inhibition of the renal renin–angiotensin system might play a role in the renal protective effects of exercise training by reducing the ROS generation in Nx-induced CRF rats.

Nx also increased renal XO activity and expression, which were effectively inhibited by exercise training. To our knowledge, there are no reports that exercise training affects XO in the kidneys of Nx-induced CRF rats. Previous studies have indicated that XO inhibitors improve renal dysfunction in various CRF models [33,34] as well as in Nx-induced CRF rats [16]. Thus, we surmise that the exercise training-reduced XO might ameliorate renal oxidative stress in Nx-induced CRF rats.

We have reported that exercise training ameliorates renal oxidative stress and ROS generation enzymes in several hypertensive rat models. In spontaneously hypertensive rats, exercise training with treadmill running for 8 weeks decreased blood pressure, urinary MDA, and renal NADPH oxidase activity but did not change renal XO activity [35]. In Dahl salt-sensitive rats fed a high salt diet, exercise training with treadmill running for 8 weeks decreased renal XO activity but did not affect blood pressure or renal NADPH oxidase activity [36]. Pharmacological studies reported the effects of ROS generation inhibitor on blood pressure and renal dysfunction in Nx-induced CRF rats. The superoxide dismutase mimetic, Tempol, decreased renal oxidative stress but did not affect proteinuria, renal function, or glomerular and tubulointerstitial damage [12]. The NADPH oxidase inhibitor, apocynin, lowered blood pressure but did not affect serum creatinine in Nx-induced CRF rats [13]. The XO inhibitor, febuxostat, improved proteinuria, renal dysfunction, and renal interstitial fibrosis without lowering blood pressure in Nx-induced CRF rats [16]. The results of the pharmacological studies indicate that the NADPH oxidase and XO-induced ROS generation induces blood pressure elevation and renal damages, respectively, in Nx-induced CRF rats. The reduction of NADPH oxidase and XO activities might be involved in the antihypertensive and renal protective effects of exercise training in Nx-induced CRF rats.

Some studies have reported the effects of exercise on oxidative stress in humans. Intradialytic exercise training for 6 months decreased serum MDA and increased antioxidant capacity and catalase levels in dialysis patients [37]. Exercise training for 4 months ameliorated levels of the plasma oxidative stress marker, F2-isoprostane, in CKD patients [38]. Thus, the antioxidant effects of exercise training may be comparable between patients and rats with CKD or CRF.

A limitation of this study is that effects of exercise training on the ROS scavenging system were not examined. Exercise training with treadmill running for 8 weeks before performing the Nx operation increased antioxidant enzymes, such as superoxide dismutase and catalase, but did not ameliorate renal dysfunction in Nx-induced CRF rats [39]. Furthermore, exercise training with treadmill running for 8 weeks increased superoxide dismutase and glutathione peroxidase activities but not catalase activity in the kidney of Nx-induced CRF rats, but exercise training did not ameliorate renal dysfunction [40]. These results suggest that the ROS scavenging may be not involved in the renal protective effects of exercise training in Nx-induced CRF rats. In contrast to the previous study, our study showed that exercise training for 12 weeks attenuated not only the NADPH oxidase and XO-induced ROS generation but also hypertension and renal dysfunction in Nx-induced CRF rats. It is possible that the renal protective effect of exercise training may depend on experimental models and exercise protocols.

In conclusion, exercise training attenuates hypertension and renal dysfunction and ameliorates renal oxidative stress and ROS generation enzymes, NADPH oxidase and XO, in Nx-induced CRF rats. The reduction of the NADPH oxidase and XO-induced ROS generation may be involved in the renal protective effects of exercise training.

## Figures and Tables

**Table 1:** Effects of exercise training on body weight, systolic blood pressure, and plasma and urinary parameters.

	Sham	NxS	NxEx
Body weight (g)	508.0 ± 7.6	397.8 ± 42.6 *	444.0 ± 11.7
Systolic blood pressure (mmHg)	123 ± 2	203 ± 5 **	165 ± 2 **^††^
Plasma creatinine (mg/dL)	0.2 ± 0.01	2.1 ± 0.5 **	0.7 ± 0.5 ^††^
Plasma urea nitrogen (mg/dL)	17.0 ± 0.3	161.9 ± 39.8 **	48.9 ± 3.5 ^††^
Urinary protein (mg/day)	9.3 ± 1.5	295.7 ± 33.2 **	175.0 ± 10.5 **^††^

Sham operation, Sham; Nephrectomy sedentary, NxS; Nephrectomy exercise, NxEx. Values are the means ± SEM. * *p* < 0.05, ** *p* < 0.01 compared with the sham group, ^††^ *p* < 0.01 compared with the NxS group.

## Data Availability

The data that support this study are available from the corresponding author upon reasonable request and with permission of all authors.

## Abbreviations

CKD: chronic kidney disease; CRF, chronic renal failure; Nx, 5/6 nephrectomy; reactive oxygen species; ROS, nicotinamide adenine dinucleotide phosphate; NADPH, xanthine oxidase; XO, MDA; malondialdehyde, TBARS; thiobarbituric acid reactive substances.
